# The Relationship between Bariatric Food Pyramid and Long-Term Anthropometric Measurements of Patients Undergoing Bariatric Surgery

**DOI:** 10.1155/2022/8291512

**Published:** 2022-05-30

**Authors:** Nihal Zekiye Erdem, Fatma Mert-Biberoğlu, Halit Eren Taşkın

**Affiliations:** ^1^Istanbul Medipol University, School of Health Sciences, Department of Nutrition and Dietetic, Istanbul, Turkey; ^2^Istanbul University-Cerrahpaşa, Department of Metabolic & Bariatric Surgery, Department of General Surgery, Cerrahpaşa Medical Faculty, Istanbul, Turkey

## Abstract

The bariatric food pyramid is a standard for long-term healthy living and nutritional habits of patients who have undergone bariatric surgery, taking their gastric capacity and special nutritional requirements into account. This study aimed to evaluate how the compliance with the pyramid affects the anthropometric change in patients who have undergone bariatric surgery, depending on the period after surgery. 81 patients who have undergone bariatric surgery between August 2016 and September 2018 participated in the study. The patients were evaluated in the postoperative period and were divided into three groups according to the year they had the operation. Food consumption frequency information was obtained from the patients, and the amount of food consumed per day was recorded in grams. Protein, vegetable, fruit, grain, and oil consumption was calculated according to the pyramid and calculated as portions. A statistically significant difference was found for all three groups in terms of weight loss and body mass index (BMI) changes before and after surgery (*p* < 0.001, *p* < 0.001, respectively). It was observed that the amount of protein consumed by the patients was sufficient, cereal was high, and fruit was insufficient. Patients who consumed foods that were not recommended slowed in weight loss. In conclusion, it is estimated that increased consumption of grains and nonrecommended foods may cause weight gains. In order to prevent this, it is necessary to ensure that patients are fed in accordance with the pyramid and followed for many years.

## 1. Introduction

Bariatric surgery is currently considered the gold standard in the treatment of morbid obesity and comorbidities, when diet and exercise do not provide expected body weight losses [1, 2].

As a result of sleeve gastrectomy (SG) and gastric bypass procedures, intake, digestion, and absorption of both macronutrients and micronutrients change significantly. This may be the result of restriction of gastric capacity, malabsorption, changes in hormones such as ghrelin, glucagon-like peptide 1 (GLP-1), peptide YY (PPY) that affect appetite, or a combination of these mechanisms. Thus, important nutritional deficiencies may occur and can lead to serious complications [3].

Physiological and anatomical gastrointestinal changes that occur after surgery affect food tolerance [1, 2, 4]. If food intolerance continues after surgery, food avoidance or maladaptive behaviors may be observed [1, 5, 6]. Since these changes will cause nutritional deficiency, patients should make changes in their diet [1, 4, 7]. The main nutrients affected after surgery are vitamins A, B1, B6, B12, C, D, and E, folate, iron, zinc, selenium, and calcium. Proper nutritional therapy is essential for both successful weight loss and the reduction or prevention of nutritional deficiencies that can have serious long-term consequences [3, 8, 9].

Lifetime nutrition education and evaluation of diet are the keys to the long-term success of bariatric surgery. Some dietary guidelines for patients undergoing bariatric surgery have been published; however, these mostly focus on short-term dietary recommendations. Prevention of weight loss and undesired complications depends on lifelong regulation of eating behavior and physical activity [10].

Bariatric procedures can lead to malnutrition when proper diet is not followed [9]. American Society for Metabolic & Bariatric Surgery (ASMBS) prepared nutritional recommendations to protect the health and weight loss of patients that undergoing bariatric surgery and to reduce nutritional deficiency. These recommendations are shown in the special food pyramid created for late postoperative bariatric surgery patients [8, 11].

The bariatric food pyramid is a standard taking patients' gastric capacity and special nutritional requirement into account for their long-term healthy living and nutritional habits. ***At the bottom of the pyramid*** are water and caffeine-free beverages, vitamin-mineral supplements that should be taken every day. Also, physical activity is included here. ***In the second level of the pyramid***, there are foods with high protein content. High-protein and low-fat food options are recommended to control energy intake. ***At the third level of the pyramid*** are vegetables and fruits that are high in fiber and low in calories. ***The fourth level of the pyramid*** contains grains. Limited consumption of carbohydrates is emphasized with the recommendation of two servings of whole grains per day. ***At the top of the pyramid*** are high-calorie foods and drinks that should be avoided [8, 10].

Nutrition in accordance with the pyramid after surgery is important for achieving the desired weight loss and maintaining its continuity. This study aims to evaluate how pyramid-compatible nutrition affects weight change depending on the postoperative period in patients that undergoing bariatric surgery.

## 2. Methods and Materials

The study was approved by Non-Invasive Research Ethics Committee with the number 10840098–604.01.01-E.12080, decision number 246, and date 09.03.2020, and verbal consent was obtained from each participant upon informing by phone.

### 2.1. Features of Participants

This study was carried out with 81 patients (49 women, 32 men) aged 18–65 who have undergone bariatric surgery between August 2016 and September 2018. Of the 640 patients interviewed for the study, 81 voluntarily participated in the study.

### 2.2. Inclusion Criteria in the Study

Patients with BMI above ≥40 kg/m^2^ in the preoperative period and patients with BMI between 35 and 40 kg/m^2^ and having at least two of the comorbidities specified in the ASMBS guideline were included in the study [9, 11].

### 2.3. Exclusion Criteria in the Study

1. In the preoperative period if there is any of the following: anesthesia or surgery risk, mental retardation, severe unstable psychotic disorders, alcohol and drug addiction, short-term life-threatening disease (such as cancer), gastrointestinal inflammatory bowel diseases, upper gastrointestinal bleeding, kidney disease, and various malabsorption disorders.
2. In the postoperative period if there is any of the following: anastomotic leakage, patients with sepsis and staying in intensive care, severe unstable psychotic disorders, upper gastrointestinal bleeding, gastrointestinal inflammatory bowel diseases.

### 2.4. Patient Selection and Grouping

The patients participating in the study were divided into three groups according to the time passed since their surgery at the time of the study: 1st year (*n* = 27) within the first 12 months of postoperative, 2nd year (*n* = 27) between 12 months and 24 months, and 3rd year (*n* = 27) between 24 and 36 months. Roux-en-Y gastric bypass and one anastomosis gastric bypass surgeries were examined under the title of gastric bypass.

### 2.5. Study Design

Demographic information and anthropometric measurements (height, weight) of the patients were taken retrospectively from the patient registry system. All the patients have been accepted to surgery after an approval from a bariatric surgery council of the university hospital. This council consists of an endocrinologist, bariatric surgeon, bariatric nutritionist, psychologist, and exercise physiotherapist. Eating behavior of the patients was carefully monitored for at least 3 weeks by the nutritionist and the psychologist, and the surgical decision is made according to the suggestions of all the members. A postoperative gastroscopy was done by the bariatric surgeon to rule out any gastric malignancies. Routine bloodwork including lipid profile, complete blood count, liver, renal function tests, fasting glucose, and coagulation parameters was ordered preoperatively and checked by the team. Also, liver sonography was also ordered to see if the patient has a hepatosteatosis or not. Patients have been followed up at the 1st, 3rd, 6th, and 12th months and yearly after. All the patients are being followed by the same team who prepares the patient for the surgery. In our center, postoperative follow-up rate is 74% and we try to increase this rate by connecting to patients via routine calls and SMS and online support meetings. We do online support group meetings every month for our patients. There is no mortality in our patient series. Overall postoperative complication rate is 4% including mostly minor complications. Our re-hospitalization rate is less than 2%. All the patient data are routinely shared with IFSO Center of excellence database and monitored.

Each patient was called by phone, and frequency of food consumption for the last month was obtained. The quantities of foods whose consumption is questioned in the pyramid are based on patient statements. Consumption frequency and 1-day food consumption also represent a cross section. It reflects the daily consumption of the person close to reality. Therefore, this method was used [12].

The amount of food consumed per day was recorded as grams. Protein, vegetable, fruit, grain, and oil consumption was calculated according to the bariatric food pyramid and calculated as portions.

### 2.6. Statistical Evaluation

All analyses were performed using IBM SPSS Statistics 25.0 package program. Continuous variables were evaluated with mean ± standard deviation. The normality of the distribution of variables was demonstrated with the Kolmogorov–Smirnov test. Comparison between the two quantitative groups of variables was made using Student's *t*-test for independent samples. When comparing more than two groups, nonparametric Kruskal–Wallis test was used. The relationship between the two variables was investigated with Pearson's correlation coefficient (*r*) and Spearman's correlation coefficient (rs). *p* values of less than 0.05 were regarded as statistically significant.

## 3. Results

81 people with an average age of 42.98 ± 10.66, of which 39.51% was male and 60.49% was female, were included in the study.  Table 1 includes demographic and anthropometric information of the participants.

A statistically significant difference was found in body %total weight loss (%TWL) and BMI changes in the postoperative 1st, 2nd, and 3rd years (respectively, *p* < 0.001, *p* < 0.001) (Table 2).

When the food consumption of the patients and the portion amounts in the pyramid are compared in Table 3, it was seen that protein consumption for all three periods is at the required levels. It was observed that vegetable consumption in the postoperative second year and fruit consumption in all periods were below normal values. Vegetable oil consumption was above the normal value in the first postoperative year, while it was normal in other periods. Cereal consumption was above the normal value in all periods. In the pyramid, it was determined that foods that should be avoided such as sugar, alcohol, nuts, fruit juice, and carbonated drinks were consumed by the patients. There was no significant difference between the periods of all such foods consumed (*p* > 0.05).

When the portion amounts of the foods consumed were examined in relation to the pyramid (Table 4), patients' consumption of protein was 38.27%, vegetable was 59.26%, fruit was 91.36%, and vegetable oil was 44.44% below the recommended amount, while cereal consumption was 71.60% above the recommended amount.

When the food consumption of all patients participating in the study and excess weight loss percentage (EWL%), BMI change, and %TWL are examined (Table 5), there is a significantly negative weak correlation between the decrease in BMI and the consumption of fat and sugar (respectively *p*=0.029, *p*=0.049).

## 4. Discussion

It was determined that the majority of the patients participating were women (60.49%), and this is in parallel with other studies [2, 4, 8, 13–15]. This indicates that women are more prone to obesity as a result of their diet rich in carbohydrates and insufficient protein. It is also known that women go to hospitals more often than men for bariatric surgery [8, 13, 15].

In an article compiling 34 articles, examining the relationship between bariatric surgery and diet quality, it was stated that almost all patients had anthropometric changes [13]. When Table 2 is analyzed, the preoperative body weight and BMI values were found to be *p* < 0.001 when the statistics were made according to the postoperative values. This shows that there is a significant difference between the values before and after surgery. This situation is in parallel with other studies [2, 8, 16–21].

Excess weight loss percentage is often used to report weight loss after bariatric surgery [5]. For weight loss after bariatric surgery to be considered successful, the EWL must be 50% and above [22, 23]. In our study, it was determined that the observed weight loss was quite successful because the EWL>85% in all three periods. Similar to our study, BMI and EWL% were similar in the first year in SG patients, and losses decreased in the 5th year [19]. Similarly, in the study of Soares et al. [8] it was observed to be EWL ≥ 50%. In addition, it was stated that their BMI decreased within one year after surgery.

In the literature, it is suggested that EWL% of those who prefer protein and carbohydrate consumption to fat and protein consumption is higher [17]. In our study, when the protein, carbohydrate, and fat consumption of the patients was examined in terms of portions, it was observed that the consumption of protein was at the recommended level, cereal was more than the recommended level, and oil was at the recommended level (more than the recommended level in the 1st year). This situation can explain the high EWL%.

When the changes in BMI are examined (Table 2), it is seen that patients in all three postoperative periods declined from the morbid obesity level to the overweight category, but still have not reached their ideal weight. In the study of Bryant et al., it was shown that adherence with diet was higher in the first year, but weight gains began 12–24 months after surgery [17]. Contrary to this study, weight gain was not observed even in our patients who were in the 3rd postoperative year, but it is thought that weight gain may occur later due to slowing weight loss. The portion of cereal consumed by 71.60% of the patients is more than recommended. They also consume saturated, trans-fat, high-calorie, energy-dense foods and beverages at the top of the pyramid and should avoid them. Alas, if they continue to eat like this, it is thought that weight gain will be faster. In this context, one study showed an increase in TWL% with decreased pleasure and desire for foods and beverages (especially those with high fat and carbohydrate density) that are recommended to be reduced or avoided after surgery [24]. In addition, the guideline issued by ASMBS in 2020 reported that in cases where carbohydrate consumption is too high, many patients regain more than one-third of their weight loss [3]. This shows how important it is to limit carbohydrate consumption in patients who have undergone bariatric surgery.

It is already well established that dietary adherence following bariatric surgery is associated with weight changes, and that excessive intake of high-energy-dense foods and simple carbohydrates can lead to both dumping symptoms (after Roux-en-Y gastric bypass (RYGB)) and long-term postoperative weight gain [8]. However, the seriousness of this issue for patients is not well understood. For this reason, we will contribute to the literature with this study, by sharing the results of studies conducted in this field both in the world and in our country, with patients, to reveal the seriousness of the situation, to emphasize the importance of follow-up and nutrition with the bariatric team after surgery.

In Table 3, consumption amounts are compared with the recommended food portions in the pyramid. In all the three periods, cereal consumption limited to two portions is above the recommended portions and is in parallel to other similar studies [2, 8, 15, 16]. Protein portions are at the recommended level in patients in all three periods and are similar to other studies [2, 16] except for one study [8]. Fruit portions are lower than recommended for patients in all three periods and are similar to other studies [8, 15, 16]. It is not recommended to consume sugars, oils, and carbonated and alcoholic beverages that are at the top of the pyramid [8]. In particular, the consumption of these foods will cause excess energy intake, and there will be an increase in weight in patients [8, 24]. In studies after gastric bypass and SG, it was determined that the consumption of fatty foods, sweets, nonalcoholic and alcoholic beverages, and snacks decreased very much and was compatible with the pyramid, emphasizing the importance of dietitian counseling [19, 21, 25].

Sugary foods are highly osmotic and may cause dumping syndrome, and gastrointestinal complications in patients can be seen after surgery as fatty foods trigger nausea and vomiting [8, 20, 25].

When the percentage of food consumed by the patients recommended in the pyramid was examined (Table 4), it is observed that 38.27% of the patients' protein, 59.26% vegetable, 91.36% fruit, and 44.44% vegetable oil consumption was below the recommended amount and 71.60% was above the recommended amount in terms of cereal consumption. This insufficient nutritional habit observed in the vast majority of patients will cause vitamin-mineral deficiency. This situation will further increase the level of vitamin-mineral deficiency after surgery.

It has been reported that 2 years after gastric bypass, a decrease in fat, sugar, and carbohydrate intake and food choices with low energy density lead to increased protein intake and therefore weight loss [26]. Similarly, Table 5 shows that there is a significant negative and weak correlation between the decrease in BMI and the consumption of fat and sugar in our study. In addition, a negative, although not significant, correlation was observed between EWL%, BMI, and %TWL, and the foods and beverages that are not recommended. Therefore, food and drinks at the upper level of the pyramid are not recommended for patients who have undergone bariatric surgery.

We are still following the patients who participated in the study. We will evaluate the status of our patients who have completed the first five years after giving nutrition education.

Since we did not question the level of physical activity in our study, we could not comment on whether the change in EWL% and BMI levels was due to nutrition or physical activity. This constituted the limitation of our study. Additionally, it was not questioned whether the patients took vitamin-mineral supplements, but according to the guidelines, patients were informed and prescribed that they should take vitamin supplements after surgery.

In the studies, only the food groups that should be consumed by the patients were evaluated. We also included foods and beverages to avoid in our study. As a result of our study, it was determined that they were consumed by the patients and their amounts were determined. The determination of this situation will be important for the follow-up of the patients. This shows the strength of our study.

In this study, how the nutrition according to the pyramid affects the anthropometric change in patients who have undergone bariatric surgery depending on the time elapsed after the surgery was investigated. It was observed that protein consumption was sufficient, cereal consumption was high, fruit consumption was insufficient, foods that were not recommended to be consumed were consumed by the patients, and consequently weight loss slowed down and even weight gains could begin. In order to prevent this, the first recommendations to be made to patients after surgery should be made by considering the bariatric food pyramid and their nutrition should be followed for many years.

## Figures and Tables

**Table 1:** Demographic and anthropometric characteristics of the studied population.

	Participants (*n* = 81)
Gender	
Female	49 (60.49%)
Male	32 (39.51%)
Age	42.98 ± 10.66
Operation type	
-Sleeve gastrectomy	40 (49.38%)
-Gastric bypass	41 (50.62%)
Body weight (kg)	120.05 ± 25.57
BMI (kg/m^2^)	43.63 ± 10.99

Data are expressed as average ± SD. BMI : body mass index.

**Table 2:** Preoperative and postoperative changes in body weight, BMI, and % excess weight loss.

	1st year (*n* = 27)			2nd year (*n* = 27)			3rd year (*n* = 27)		
	Pre-op	Post-op	^*∗*^ *p* value	Pre-op	Post-op	^*∗*^ *p* value	Pre-op	Post-op	^*∗*^ *p* value
Gender									
Female	14 (51.85%)			18 (66.67%)			17 (62.96%)		
Male	13 (48.15%)			9 (33.33%)			10 (37.04%)		
Age (years)	40.41 ± 11.94			45.93 ± 10.82			42.59 ± 8.60		
Operation type—sleeve	16 (59.26%)			7 (25.93%)			17 (62.96%)		
gastrectomy—gastric bypass	11 (40.74%)			20 (74.07%)			10 (37.04%)		
Body weight (kg)	119.96 ± 17.63	77.70 ± 11.16	<0.001	115.67 ± 24.00	74.96 ± 13.41	<0.001	124.52 ± 32.94	75.94 ± 18.91	<0.001
BMI (kg/m^2^)	42.42 ± 6.19	27.41 ± 3.41	<0.001	41.54 ± 8.66	27.30 ± 5.14	<0.001	46.94 ± 15.52	28.49 ± 7.44	<0.001
Total weight loss (%TWL)^*∗∗*^	34.70 ± 8.33			33.59 ± 13.82			36.75 ± 16.17		
EWL (%)^*∗∗∗*^	87.85 ± 17.90			95.95 ± 50.83			86.72 ± 36.11		

Data are expressed as average ± SD. BMI : body mass index, EWL : excess weight loss. ^*∗*^Paired sample *t*-test. ^*∗∗*^*p*=0.464 (Kruskal–Wallis test). ^*∗∗∗*^*p*=0.0.782 (Kruskal–Wallis test).

**Table 3:** Daily intake of food group servings by patients after bariatric surgery.

Food groups (recommended servings)	1st year (*n* = 27)	2nd year (*n* = 27)	3rd year (*n* = 27)	*p*
Protein (4–6) Minimum to maximum	5.35 ± 2.14 1.71–9.26	4.37 ± 2.02 0.83–9.66	4.75 ± 2.38 1.37–10.26	0.256
Vegetables (2–3) Minimum to maximum	2.13 ± 1.70 0.71–9.72	1.86 ± 1.01 0.16–4.20	2.36 ± 1.73 0.54–7.40	0.706
Fruits (2–3) Minimum to maximum	0.54 ± 0.59 0.00–2.14	0.56 ± 0.60 0.00–2.14	0.69 ± 0.85 0.00–3.21	0.837
Vegetable oil (2–3) Minimum to maximum	3.21 ± 2.40 0.00–10.57	2.95 ± 2.27 0.00–8.83	2.57 ± 2.66 0.00–13.00	0.407
Grains and cereals (2) Minimum to maximum	3.48 ± 2.98 0.22–10.40	4.30 ± 2.45 0.48–9.15	4.16 ± 3.83 0.26–17.16	0.269
Sugar (avoid) Minimum to maximum	13.89 ± 11.88 0.00–38.57	15.67 ± 11.36 1.00–45.00	16.89 ± 15.52 0.00–49.86	0.720
Alcoholic beverages (avoid) Minimum to maximum	5.48 ± 13.04 0.00–42.00	6.77 ± 22.85 0.00–110.30	12.76 ± 44.94 0.00–216.00	0.864
Nuts (avoid) Minimum to maximum	23.48 ± 23.57 0.00–80.00	36.14 ± 47.09 1.00–231.00	29.22 ± 35.94 0.00–150.00	0.565
Juice (avoid) Minimum to maximum	29.48 ± 76.98 0.00–400.00	50.22 ± 94.19 0.00–400.00	28.00 ± 62.62 0.00–200.00	0.236
Carbonated beverages (avoid) Minimum to maximum	67.11 ± 232.78 0.00–1200.00	51.48 ± 194.16 0.00–1000.00	8.15 ± 23.11 0.00–84.00	0.396

Data are expressed as average ± SD. p: Kruskal–Wallis test.

**Table 4:** Comparison of consumed food groups using bariatric food pyramid recommendations.

Food groups (recommended servings)	1st year (*n* = 27)	2nd year (*n* = 27)	3rd year (*n* = 27)	All (*n* = 81)
Protein <4 portions 4–6 portions >6 portions	7 (25.93%) 10 (37.04%) 10 (37.04%)	13 (48.15%) 10 (37.04%) 4 (14.81%)	11 (40.74%) 9 (33.33%) 7 (25.93%)	31 (38.27%) 29 (35.80%) 21 (25.93%)
Vegetables <2 portions 2–3 portions >3 portions	16 (59.26%) 7 (25.93%) 4 (14.81%)	17 (62.96%) 7 (25.93%) 3 (11.11%)	15 (55.56%) 6 (22.22%) 6 (22.22%)	48 (59.26%) 20 (24.69%) 13 (16.05%)
Fruits <2 portions 2–3 portions >3 portions	25 (92.59%) 2 (7.41%) 0 (0.00%)	25 (92.59%) 2 (7.41%) 0 (0.00%)	24 (88.89%) 2 (7.41%) 1 (3.70%)	74 (91.36%) 6 (7.41%) 1 (1.23%)
Oil <2 portions 2–3 portions >3 portions	10 (37.04%) 5 (18.52%) 12 (44.44%)	13 (48.15%) 2 (7.41%) 12 (44.44%)	13 (48.15%) 9 (33.33%) 5 (18.52%)	36 (44.44%) 16 (19.75%) 29 (35.80%)
Grains and cereals ≤2 portions >2 portions	11 (40.74%) 16 (59.26%)	5 (18.52%) 22 (81.48%)	7 (25.93%) 20 (74.07%)	23 (28.40%) 58 (71.60%)

**Table 5:** Correlation of EWL%, BMI change, and %TWL with food groups

	EWL% (*n* = 81)		BMI change (*n* = 81)		%TWL (*n* = 81)	
	*r*	*p*	*r*	*p*	*r*	*p*
Protein	0.040	0.724	−0.038	0.734	0.040	0.724
Vegetables	−0.090	0.424	−0.112	0.321	−0.090	0.424
Fruits	0.010	0.928	0.077	0.492	0.010	0.928
Oil	−0.111	0.322	−0.**242**^*∗*^	0.**029**	−0.111	0.322
Grains and cereals	−0.134	0.234	−0.087	0.438	−0.134	0.234
Sugar	−0.158	0.160	−0.**219**^*∗*^	0.**049**	−0.158	0.160
Nuts	0.084	0.457	0.060	0.593	0.084	0.457
Juice	−0.113	0.315	−0.153	0.172	−0.113	0.315
Carbonated beverage	−0.130	0.248	−0.132	0.240	−0.130	0.248

^*∗*^Spearman correlation.

## Data Availability

The data used to support the findings of this study are available from the corresponding author upon request.
